# Novel β-lactamase inhibitors: a therapeutic hope against the scourge of multidrug resistance

**DOI:** 10.3389/fmicb.2013.00392

**Published:** 2013-12-24

**Authors:** Richard R. Watkins, Krisztina M. Papp-Wallace, Sarah M. Drawz, Robert A. Bonomo

**Affiliations:** ^1^Department of Internal Medicine, Northeast Ohio Medical UniversityRootstown, OH, USA; ^2^Division of Infectious Diseases, Akron General Medical CenterAkron, OH, USA; ^3^Research Service, Louis Stokes Cleveland Department of Veterans Affairs Medical CenterCleveland, OH, USA; ^4^Department of Medicine, Case Western Reserve UniversityCleveland, OH, USA; ^5^Department of Lab Medicine and Pathology, University of MinnesotaMinneapolis, MN, USA; ^6^Pharmacology, Case Western Reserve UniversityCleveland, OH, USA; ^7^Molecular Biology and Microbiology, Case Western Reserve UniversityCleveland, OH, USA

**Keywords:** antibiotic resistance, β-lactamase inhibitors

## Abstract

The increasing incidence and prevalence of multi-drug resistance (MDR) among contemporary Gram-negative bacteria represents a significant threat to human health. Since their discovery, β-lactam antibiotics have been a major component of the armamentarium against these serious pathogens. Unfortunately, a wide range of β-lactamase enzymes have emerged that are capable of inactivating these powerful drugs. In the past 30 years, a major advancement in the battle against microbes has been the development of β-lactamase inhibitors, which restore the efficacy of β-lactam antibiotics (e.g., ampicillin/sulbactam, amoxicillin/clavulanate, ticarcillin/clavulanate, and piperacillin/tazobactam). Unfortunately, many newly discovered β-lactamases are not inactivated by currently available inhibitors. Is there hope? For the first time in many years, we can anticipate the development and introduction into clinical practice of novel inhibitors. Although these inhibitors may still not be effective for all β-lactamases, their introduction is still welcome. This review focuses on the novel β-lactamase inhibitors that are closest to being introduced in the clinic.

## INTRODUCTION

The ongoing dissemination of multi-drug resistant (MDR) bacteria is a serious threat to global health. Microbiological and epidemiological surveys commissioned by public and private institutions paint a frightening portrait of the emergence of β-lactam resistance in both the community and the hospital setting. A major mechanism for antibiotic resistance among Gram-negative bacteria is the production of β-lactamases. β-lactamases are enzymes that inactivate β-lactam antibiotics by hydrolyzing the amide bond of the β-lactam ring. β-lactamases are bacterial resistance determinants that have been known for more than seventy years, yet the details of their evolution, dissemination and hydrolytic capacity still remains a great scientific challenge.

Two classification systems are presently used to categorize β-lactamases. Introduced more than thirty years ago, the Ambler classification system divides β-lactamases into four classes (A, B, C, and D) based on their amino acid sequences (Ambler, 1980). The Bush-Medeiros-Jacoby classification system groups β-lactamases according to functional properties; this classification system uses substrate and inhibitor profiles in an attempt to organize the enzymes in ways that can be correlated with their phenotype in clinical isolates (Bush and Jacoby, 2010). For purposes of simplicity in this review, we will refer to the Ambler classification system.

Class A enzymes include both plasmid-mediated and chromosomally-encoded β-lactamases that demonstrate broad-spectra (e.g., TEM-1 and SHV-1), extended-spectra (e.g., CTX-M-15), and carbapenemase activity (e.g., KPC-2). Class B enzymes are metallo-β-lactamases (MBLs) which can hydrolyze penicillins, cephalosporins, and carbapenems such as the recently described New Delhi metallo-β-lactamase (NDM-1) found in *Klebsiella pneumoniae* and *Escherichia coli* (Kumarasamy et al., 2010). Class C enzymes are cephalosporinases that are chromosomally-encoded for example the inducible *Pseudomonas aeruginosa* AmpC and P99 β-lactamase of *Enterobacter* spp., or plasmid-mediated such as CMY-2, first found in *Escherichia* *coli*. Class D enzymes have a substrate preference for oxacillin and are referred to as oxacillinases (e.g., OXA-1). Recent surveys have shown that class D enzymes are a rapidly expanding class of β-lactamases and have enzymes that can hydrolyze extended-spectrum cephalosporins (e.g., OXA-10) and carbapenems (e.g., OXA-23). Several class D enzymes are often found in non-fermenting bacteria such as *P. aeruginosa* and *Acinetobacter baumannii* and occasionally in *E*. *coli* and *K*. *pneumoniae*.

**FIGURE 1:**
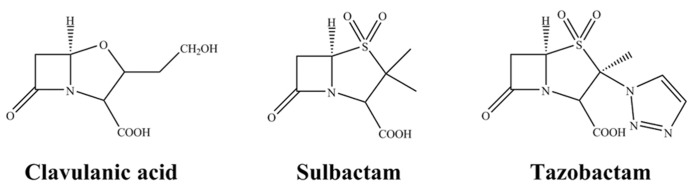
**Chemical structures of the clinically available β-lactamase inhibitors**.

At present, there are three commercially available β-lactamase inhibitors: clavulanic acid, sulbactam and tazobactam (**Figure 1**). These are mechanism-based inhibitors that share a common β-lactam structure. As a group, they are best active against most class A β-lactamases, exceptions include KPC-2 carbapenemase and inhibitor resistant TEMs (IRTs) and SHVs. Clavulanate, sulbactam, and tazobactam have less effect on class C enzymes, and are essentially inactive against class B and most class D enzymes (Bush and Jacoby, 2010).

Encouragingly, pharmaceutical companies are aggressively developing and bringing to market new combinations of β-lactam antibiotics with β-lactamase inhibitors. Several of these are now close to clinical availability. A promising new design for β-lactamase inhibitors has been to focus on scaffolds that can rapidly acylate a wide range of β-lactamases while minimizing hydrolysis. This review will focus on recent data regarding the mechanisms of inhibition of these novel agents, their antimicrobial activity, and the progress in their clinical trials. Specifically, avibactam and MK-7655 are members of a new class of non-β-lactam-β-lactamase inhibitors called diazabicyclooctanes (DBOs) with a broader spectrum of activity than other inhibitors. Recent modifications to boronic acid (BA) compounds have led to very potent *E. coli* AmpC inhibitors that are eagerly awaited. Finally, the discovery of a “universal” β-lactamase inhibitor has been an important goal of both academia and the pharmaceutical industry but has proven to be quite challenging. Emerging data show this ideal might not be feasible and researchers investigating mechanisms of β-lactamase inhibition will likely need to develop alternative strategies.

## DIAZABICYCLOOCTANES

### AVIBACTAM

**FIGURE 2:**
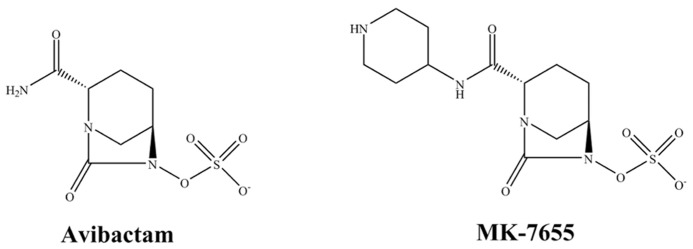
**Chemical structures of diazabicyclooctanes**.

Avibactam (AVI) is a non-β-lactam compound in the class of DBOs (**Figure 2**). As a β-lactamase inhibitor, AVI inactivates β-lactams by a reversible fast acylation and relatively slow deacylation reaction. Against most class A and class C β-lactamases this results in a low turnover ratio (Ehmann et al., 2012). The β-lactamase inhibition by AVI is mostly reversible and AVI demonstrates a half-life of 16 min for TEM-1 which closely approaches one generation time of *E. coli* (Ehmann et al., 2012). Thus despite reversibly of AVI, AVI is predicted to remain bound to TEM-1 during most of an entire generation cycle of *E*. *coli*; thus keeping the enzyme inactive. Unlike clavulanic acid and like sulbactam, AVI does not induce β-lactamase production (Coleman, 2011). In addition to TEM-1 and SHV-1, clinically important β-lactamases that are readily inhibited by AVI include the serine carbapenemase KPC-2, the ESBL CTX-M-15, class C β-lactamases such as the AmpC and some class D enzymes (OXA-48).

An interesting development is the combination of this DBO inhibitor with a number of β-lactam antibiotics that have traditionally been used to treat Gram-negative bacteria. Despite the tendency of this class of antibiotics to select ESBLs, expanded-spectrum cephalosporins are seen as potential partners because they have a broader spectrum of activity. As a result, the combination of ceftazidime-AVI has potent activity against *K. pneumoniae* carrying ESBLs such as SHV-5, other ESBLs and AmpC enzymes and also against most *Klebsiella* spp. harboring the KPC enzyme (Livermore et al., 2011). Against *P*. *aeruginosa*, AVI reverses AmpC-mediated ceftazidime resistance, reducing MICs for fully derepressed mutants and isolates to ≤8 mg/L (Mushtaq et al., 2010). Unfortunately, ceftazidime-AVI lacks activity against *A. baumannii* and most species of anaerobic bacteria (Citron et al., 2011; Zhanel et al., 2013).

Emerging data from clinical trials that are registered show that ceftazidime-AVI is as effective as carbapenem therapy for complicated urinary tract infections (UTIs) and complicated intra-abdominal infections (cIAI), including those caused by expanded-spectrum cephalosporin-resistant Gram-negative organisms (Zhanel et al., 2013). Furthermore, a recent trial of ceftazidime-AVI plus metronidazole in the treatment of cIAIs found a favorable clinical response rate when compared to meropenem (Lucasti et al., 2013).

Ceftaroline is a novel semisynthetic anti-methicillin-resistant *Staphylococcus aureus* (MRSA) cephalosporin with broad-spectrum activity. The combination of ceftaroline-AVI is active against *Enterobacteriaceae* that produce KPC, various ESBLs (CTX-M types), and AmpC (chromosomally derepressed or plasmid-mediated enzymes), as well as against those producing more than one of these β-lactamase types (Castanheira et al., 2012b). However, ceftaroline’s activity against *Acinetobacter* spp. and *P*. *aeruginosa* is limited. In a clinical study of diabetic foot infections (which are often polymicrobial), ceftaroline-AVI reduced ceftaroline MICs for strains of resistant *Enterobacter* spp. and one strain of *Morganella*, as well as for the anaerobes *Bacteroides fragilis* and *Prevotella* spp. (Goldstein et al., 2013a). A Phase 2 clinical trial comparing ceftaroline-AVI to doripenem in adults with complicated UTIs is in progress^1^

Monobactams resist hydrolysis by MBLs, thus another promising partner for AVI is aztreonam. For example, if any *Enterbacteriaceae* and *P.* *aeruginosa* strains carrying MBLs and co-produce ESBLs or AmpC, the aztreonam would target the MBLs, while the avibactam would inhibit the ESBLs and AmpC (Livermore et al., 2011; Crandon et al., 2012). As such, this combination will be a very welcome addition to the antibiotic formulary as the safety and efficacy of aztreonam are already established in clinical practice.

### MK-7655

MK-7655, a novel DBO that is structurally similar to AVI except for an additional piperidine ring, exhibits synergy in combination with imipenem against KPC-producing *K*. *pneumoniae* and *P. aeruginosa* expressing AmpC (**Figure 2**; Mangion et al., 2011; Hirsch et al., 2012). Studies show that at a concentration of 4 mg/L, MK-7655 lowers imipenem MICs for *Enterobacteriaceae* with KPC carbapenemases from 16–64 mg/L to 0.12–1 mg/L (Livermore et al., 2013). Interestingly, synergy is also seen for *Enterobacteriaceae* with carbapenem resistance mediated by porin loss. Among strains of *P.* *aeruginosa*, 4 mg/L of MK-7655 reduces the MIC of imipenem for all isolates, except those with MBLs.

Two separate Phase 2 clinical trials of two doses (125 mg or 250 mg) of MK-7655 plus imipenem-cilastatin vs. imipenem-cilastatin alone for treatment of complicated UTIs or cIAIs began in early 2012^1^. Results from these trials are eagerly awaited.

## BAs

The inhibitory effects of BAs on β-lactamases have been known for several decades. Boron forms a reversible bond with β-lactamases. Recent studies have shown that different BAs are high affinity inhibitors of the AmpC β-lactamase of *E. coli*, class A β-lactamases TEM-1, CTX-M, and SHV-1, and class C β-lactamase, ADC-7 from *Acinetobacter* spp. and *P. aeruginosa* (Drawz et al., 2010a; Winkler et al., 2013). Many BAs are in early developmental stages, however the progress of these compounds is rapidly advancing.

**FIGURE 3:**
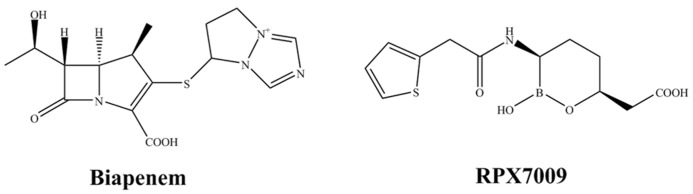
**Chemical structures of a novel combination: a carbapenem (left) with a new boron-based β-lactamase inhibitor (right)**.

Despite the large number of BAs in development, only one so far is approaching clinical trials. First introduced at the 2012 Interscience Conference on Antimicrobial Agents and Chemotherapy, RPX7009 is a new boron-based inhibitor being developed in combination with biapenem (RPX2003; **Figure 3**; Castanheira et al., 2012a; Hecker et al., 2012; Sabet et al., 2012). RPX7009 lacks direct antibacterial activity but it does enhance the activity of biapenem against class A carbapenemase-producing *Enterobacteriaceae* (e.g., KPC, SME, or IMI/NMC-A; Livermore and Mushtaq, 2013). Moreover, RPX7009 lowers the MICs of biapenem against *Enterobacteriaceae* with complex β-lactamase backgrounds (AmpC or ESBL activity) and porin losses. Unfortunately, RPX7009 does not inhibit class B MBLs and class D carbapenemases. Against *Bacteroides* and other select anaerobes, biapenem and RPX7009 demonstrates comparable activity to meropenem alone (Goldstein et al., 2013b). Regarding other anaerobes (*Fusobacterium* spp and *Prevotella*) biapenem and RPX7009 are reasonable active. *Clostridia* are a notable exception with the range extending up to 8 mg/L. As expected against MBL-producing *Bacteroides*, activity is poor.

## BAL30072 AND BAL30376

**FIGURE 4:**
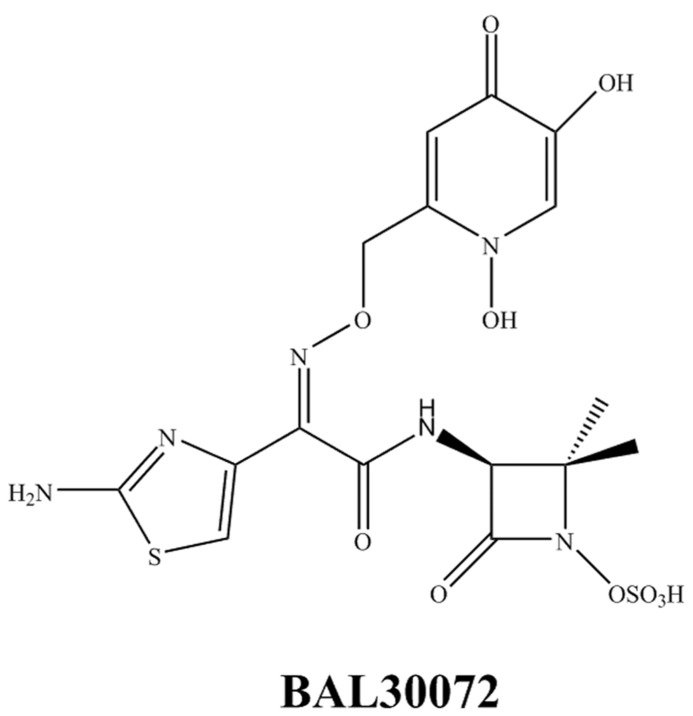
**Chemical structure of a novel siderophore monosulfactam**.

BAL30072 (**Figure 4**) is a novel siderophore monosulfactam similar to aztreonam. BAL30072 demonstrates activity against a broad range of Gram-negative bacilli including *Acinetobacter* spp*., P. aeruginosa, Burkholderia cepacia*, and some MDR *Enterobacteriaceae* (Page et al., 2010; Russo et al., 2011; Higgins et al., 2012). BAL30072 shows potency against carbapenem-resistant *Enterobacteriaceae* including those with AmpC, ESBL, and KPC enzymes, *P. aeruginosa* including most strains with MBLs and most isolates of *A. baumannii* except those producing OXA-58 (Mushtaq et al., 2013). However, resistance is still observed with the *K. pneumoniae* ST258 isolates carrying KPC. The addition of meropenem to BAL30072 increases activity against certain individual isolates of *A. baumannii*. BAL30072 is currently in a Phase 1 study and will likely be combined with meropenem in future clinical development^1^.

In addition to BAL30072, researchers have also developed another compound with broad activity against β-lactamases called BAL30376, which combines three β-lactams: the siderophore monobactam BAL19764, the bridged monobactam class C β-lactamase inhibitor BAL29880 for class C cephalosporinases, and clavulanic acid to inhibit class A enzymes (Bush and Macielag, 2010). Further *in vitro* analysis and animal studies of BAL30376 will be necessary before its developmental pathway is clear.

## NEW CARBAPENEMS AND BEYOND

Originally developed in the 1970s, carbapenems are among the most broad-spectrum antibiotics in clinical use. One major advantage of this class of agents is their stability against hydrolysis by many ESBLs and class C cephalosporinases. The unique property of carbapenems that merits their inclusion in this review is their ability to inhibit both class A and class C β-lactamases (Drawz and Bonomo, 2010; Papp-Wallace et al., 2011) and their high affinity for the bacterial transpeptidases and carboxypeptidases that synthesize the peptidoglycan-based cell wall. The carbapenem class of β-lactams act as a “slow substrates.” Crystallographic analyses show how these compounds inactivate the serine-based class A and C enzymes by adopting unique conformations in the active site that disfavor hydrolysis (carbonyl oxygen outside of the oxyanion hole). The remaining parts of this section will examine the promise of some of these carbapenems that are apart from imipenem, meropenem, ertapenem, and doripenem.

Biapenem has been available in Japan since 2002 and is currently in Phase 2 clinical study in the USA. Biapenem achieves high concentration in respiratory tissue making it an attractive choice for pulmonary infections (Bassetti et al., 2011). Biapenem is hydrolyzed by MBLs and its bicyclic derivative has significant affinity for these enzymes (Garau et al., 2005). Recent experimental evidence shows it might be possible to obtain new competitive inhibitors of B2 MBLs by modification of this bicyclic compound (Gatti, 2012).

**FIGURE 5:**
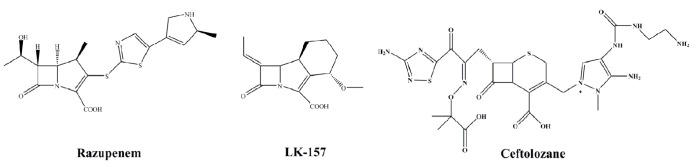
**Chemical structures of novel β-lactams with β-lactamase inhibitor activity**.

Razupenem (SMP-601; **Figure 5**) is a β-methyl carbapenem with activity against MRSA, enterococci including *Enterococcus faecium* and many species of Enterobacteriaceae. The activity of razupenem is not abrogated by ESBLs but AmpC and class A carbapenemases seem to affect it more than ertapenem or imipenem (Livermore et al., 2009). Pharmacodynamic data suggest razupenem can be dosed the same for *E. coli*, *Proteus mirabilis* and *Klebsiella* spp. as for MRSA (MacGowan et al., 2011). However, the development of razupenem has been discontinued.

LK-157 is a novel tricyclic carbapenem with potent inhibitory activity against serine β-lactamases (**Figure 5**; Plantan et al., 2007). LK-157 is a close structural analog to sanfetrinem, an oral, broad-spectrum antibiotic whose development was stopped after Phase 2 clinical trials (Babini et al., 1998). LK-157 restores the diminished activity of β-lactam antibiotics against a number of bacterial strains producing class A ESBLs (excluding CTX-M and KPC) as well as class C β-lactamases (Paukner et al., 2009). Of note, data from a rat jejunum model suggest the compound has good bioavailability, raising the exciting possibility of an oral broad-spectrum agent active against class A and C enzymes (Iglicar et al., 2009).

S-649266 is a novel cephem antibiotic that promises to be stable against MBLs^2^ Details are still forthcoming about this compound, but early reports indicate S-649266 is stable against MBL producing strains and is effective against *A. baumannii, Stenotrophomonas maltophilia,* and *Burkholderia* spp. This is not a new β-lactamase inhibitor, but the activity against MBLs merits close attention.

CXA-202 is the combination of ceftolozane (CXA-201) with tazobactam (**Figure 5**). This formulation is targeted for *P. aeruginosa* and other MDR strains and has advanced into Phase 3 trials. *Per se*, this is not a novel β-lactamase inhibitor but is a new partner. The enhanced activity of the cephalosporin partner will be examined closely as this combination represents a novel testing paradigm in this area.

## INHIBITORS OF CLASS B ENZYMES

Except for aztreonam-AVI, BAL30072, and S-649266, none of the other aforementioned experimental β-lactams and β-lactamase inhibitors have significant activity against isolates expressing MBLs. This is problematic because MBLs can spread rapidly through mobile genetic elements, as seen with the global emergence of NDM-1 (Liu et al., 2013).

The hydrolytic mechanisms of MBLs are significantly different from other classes of β-lactamases, requiring one or two zinc atoms depending on the subclass. Our understanding of MBLs is emerging as compared to the better studied class A and C enzymes (Dubus et al., 1995; Powers and Shoichet, 2002; Chen et al., 2006, 2009; Fisher and Mobashery, 2009). One class of agents that appear promising against MBLs is the thiol derivatives. Thiols, including the anti-hypertensive medication captopril, effectively inhibit several MBLs including NDM-1 and subclass B1, B2, and B3 enzymes (Heinz et al., 2003; King et al., 2012). Thiol compounds utilize the same mechanisms of zinc chelation and hydrolytic displacement. Additional clinical studies using these compounds in combination with antibiotics seem warranted.

## CHALLENGES OF INHIBITING CLASS D ENZYMES

Similar to MBLs in their diversity, class D β-lactamases are designated OXA-type because of their ability to hydrolyze oxacillin. Their substrate profiles range from narrow to broad-spectrum, including carbapenems (Nazik et al., 2012). At present, β-lactamase inhibitors effective against class D enzymes are not available but promising data are emerging.

Several class D enzyme inhibitors are in development. For instance, substituted penicillin sulfones demonstrate efficacy against a number of OXA enzymes including OXA-24/40, a clinically relevant enzyme found in *A. baumannii* (Bou et al., 2010; Drawz et al., 2010b). A compound in development, 4,7-dichloro-1-benzothien-2-yl sulfonylaminomethyl BA (DSABA), is the first BA-based class D enzyme inhibitor. DSABA inhibits class A and C enzymes as well and demonstrates synergy with imipenem against *A. baumannii* (Tan et al., 2010). A series of thiophenyl oxime phosphonate β-lactamase inhibitors with potency against OXA-24/40 have also been discovered (Tan et al., 2011). Of interest, one compound reduces the MIC of imipenem against a highly imipenem-resistant strain of OXA-24/40 producing *A. baumannii*.

## CHOOSING THE RIGHT PARTNER ANTIBIOTIC AND THE CHALLENGES AHEAD

Determining the ideal β-lactam for a given β-lactamase inhibitor and defining the ratio of the inhibitor to that β-lactam is a complex process. Indeed, it has been suggested that several considerations should be taken into account: (1) the ability of the inhibitor to protect the β-lactam ring from hydrolysis by key target enzymes; (2) the quantity of inhibitor needed to protect the β-lactam ring; (3) the feasibility and stability of the formulation; (4) pharmacokinetic and dosing parameters; and (5) cost (Shlaes, 2013). However, it is difficult to use standard pharmacokinetic and pharmacodynamic indices with inhibitors because they have weak to no intrinsic antimicrobial activity and they are usually partnered with an active antimicrobial agent. Mathematical modeling is one approach to these challenges. Using mathematical systems in pharmacodynamic models may help define regimens for inhibitors to prevent false labeling of a drug as ineffective because of dosing failures (Bush, 2012).

The report of a single isolate of *K. pneumoniae* producing a serine carbapenemase, a MBL, an ESBL and a plasmid-encoded AmpC carbapenemase underscores the challenge of using β-lactam antibiotics in the clinical setting (Pournaras et al., 2010). Treating this kind of pathogen with a β-lactam will likely require one with high stability to many common β-lactamases (e.g., aztreonam), together with two or more β-lactamase inhibitors that inhibit MBLs and serine β-lactamases. An example is the triple compound BAL30376 (Bush and Macielag, 2010; Livermore et al., 2010; Page et al., 2011). In addition to exerting a bactericidal effect against a wide range of β-lactamase-producing organisms including strains that were resistant to other β-lactams (except for KPC carbapenemases); BAL30376 is also relatively refractory toward selection of resistant mutants (Page et al., 2011).

## FUTURE PERSPECTIVE

The majority of the compounds reviewed in this paper are in preclinical stages and (with the exception of AVI and MK-7655) are years away from availability. Thus, the pace of drug development must increase in order to meet the Infectious Diseases Society of America’s goal of 10 new systemic drugs to treat infections caused by resistant bacteria by 2020 (Infectious Diseases Society of America, 2010; Boucher et al., 2013). The lack of drug candidates potentially active against MBLs is a great concern. For infections caused by bacteria harboring MBLs, treatment options are limited to polymyxins, tigecycline, and fosfomycin. Moreover, new β-lactamases are reported worldwide with alarming frequency, which continues to put strain on our existing antibiotic armamentarium (Lamoureaux et al., 2013). While novel β-lactamase inhibitors with new mechanisms of action provide substantial advances compared to currently available agents, incremental advances to existing classes are also valuable and should be encouraged (Page and Heim, 2009). The long quest for a universal β-lactamase inhibitor is becoming increasing quixotic with more pragmatic approaches, such as drug combinations, now a leading paradigm.

A plethora of strategies to invigorate drug development have been recently proposed (Infectious Diseases Society of America, 2012; Spellberg et al., 2013). These include conducting superiority and organism-specific clinical trials, transparency through public reporting of antibiotic usage tied to reimbursement, using molecular techniques for diagnostic confirmation of antibiotic indications, and investigating agents that modify host immune responses to pathogens to circumvent resistance selection. We also suggest that attention be given to alternative agents with activity against β-lactamases. Additional research studies are warranted especially since MBLs are important drivers of pan-resistant phenotypes. We remain positive in our outlook as the progress to date merits confidence that new drugs will be available very soon.

## Conflict of Interest Statement

The authors declare that the research was conducted in the absence of any commercial or financial relationships that could be construed as a potential conflict of interest.

## Footnotes

1 www.clinicaltrials.gov

2 www.shionogi.com/pdf/RDmeeting2012.pdf
